# Dance therapy and cognitive impairment in older people: A review of clinical data

**DOI:** 10.1590/1980-5764-DN-2021-0103

**Published:** 2022-07-29

**Authors:** Ana Clara Menezes, Gabrielle Drumond, Nadia Shigaeff

**Affiliations:** 1 Universidade Federal de Juiz de Fora, Departamento de Psicologia, Juiz de Fora MG, Brazil.; 2 Universidade Federal de Juiz de Fora, Núcleo Interdisciplinar em Pesquisa em Neuropsicologia e Gerontologia, Juiz de Fora MG, Brazil.

**Keywords:** Aged, Cognition, Dance Therapy, Cognitive Dysfunction, Dementia, Idoso, Cognição, Terapia através da Dança, Disfunção Cognitiva, Demência

## Abstract

The growing interest for nonpharmacological treatment alternatives to older people with mild cognitive impairment or dementia has increased exponentially for the past few years; in this context, dance therapy is an effective therapeutic tool in improving the cognition of older people. The aim of this study was to verify whether dance therapy is a viable tool in promoting benefits with regard to the cognition and mood of older people with cognitive impairment. A database search covering the past 10 years was carried out. Result: The search found 193 papers; after title, abstract, and duplicity analysis, 14 of those were selected, of which 10 were fully revised. The studies showed positive results regarding the improvement of cognitive function after dance stimulations, as well as beneficial effects on the mood of older people with cognitive impairment.

## RESUMO

O interesse por alternativas de tratamento não farmacológico para idosos com demência ou comprometimento cognitivo leve aumentou nos últimos anos. Entre elas, a dança destaca-se como uma eficaz ferramenta terapêutica na cognição de idosos. Dessa forma, o objetivo da presente revisão foi determinar se a dança é uma ferramenta capaz de promover benefícios em relação à cognição e ao humor de idosos comprometidos cognitivamente, por meio de uma revisão das publicações de estudos longitudinais dos últimos dez anos, que avaliaram os efeitos na cognição da terapia por intermédio da dança em idosos com alterações cognitivas, nas bases de dados PubMed, Scientific Electronic Library Online (SciELO) e Cochrane Library. A busca identificou 193 artigos; após a análise de títulos, resumos e duplicidade, 14 foram selecionados, dos quais dez foram revisados. De maneira geral, os estudos apresentaram resultados positivos quanto à melhora das funções cognitivas após a estimulação com dança, além de efeitos positivos também no humor de idosos comprometidos cognitivamente.

## INTRODUCTION

The aging process is characterized by changes in the brain and in the global performance of cognitive function. Therefore, cognitive abilities such as memory, processing speed, executive function, and reasoning seem to follow a pattern of decline during old age^1,2^. However, these cognitive declines are not necessarily accompanied by impacts on the individual’s functionality, except for some specific circumstances in which the dysfunction is considered pathological, such as in dementia.

Mild cognitive impairment (MCI) refers to a notable, but discreet, alteration in cognitive function, more prevalent during old age and that does not result in any loss of functionality^2,3^. MCI is characterized by the impairment of at least one cognitive domain and can also be defined as amnesic (the most common subtype) or nonamnesic, depending on whether or not memory is compromised^4–6^. Consequently, the ability to obtain and retain new information is also significantly affected^3,5^. Historically, MCI was defined as a transitional phase between healthy aging and dementia^2,7,8^. It is relevant to note that not all MCI cases will necessarily lead to dementia, although the chances are significantly higher^5^. Indeed, in most cases, the cognitive impairment can be reverted to a performance considered typical to the respective age group^8^.

Dementia refers to the process of generalized and progressive deterioration of cognitive functions, at a level severe enough to compromise the individual’s independence and negatively impact their ability to perform daily life activities^9^. There is no specific and unique etiology to dementia; it can be caused by a number of different preexisting diseases. In fact, there are more than 100 possible causes of dementia, the most common being Alzheimer’s disease^2^. It is estimated that 7% of the world population over 65 years of age is affected by some sort of dementia, and Latin American countries are the most affected^10^.

Despite being widely studied, there is still no cure or effective treatment to reverse the symptoms of dementia once the diagnosis has been made. However, there are certain nonpharmacological interventions that can be useful in the delay of the pathology’s progression or in controlling the symptoms^5,11,12^. Among those interventions, dance therapy is a relatively recent construct, but some studies have shown it to be effective.

Dance is a universal cultural expression of the human being that crosses the time barrier, motivating and mobilizing all age groups. When music and body movements are involved, several regions of the brain are activated concomitantly, promoting the chance of neuroplasticity and other effects that are the aim of investigations by researchers in the field^13,14^.

Given the above fact, dance therapy is considered an excellent nonpharmacological intervention as it provides a body activity that is extremely advantageous together with music that favors an emotional bond, facilitating the adherence of older people to the proposed intervention^15^. Regarding the effects of dance for people with cognitive impairment, evidence of improvement in cognitive performance is found, demonstrating that dance therapy not only acts as a preventive factor for neurodegenerative diseases but is also able to promote the decrease in cognitive impairment, especially of memory^16^. Therefore, in view of some many benefits described in healthy older people or those with some kind of impairment, further investigation to clarify the impacts of dance as therapeutic intervention becomes essential.

However, when considering publications in the area, it is clear that the systematic reviews found include in their methodology studies that have a sample constituted exclusively of cognitively healthy people, in addition to other interventions where the sample had some kind of impairment; similarly, many reviews only consider a specific diagnosis in its inclusion criteria, such as Alzheimer’s disease or Parkinson’s disease. Therefore, the objective of the present review was to verify whether there are, in the literature, studies that discuss the benefits of dance therapy for the cognition and mood of older people with some degree of cognitive impairment, through the search for publications in the past 10 years. Furthermore, this review also aimed to understand the effect of dance therapy, as a therapeutic tool, for this population, as well as to be able to point out which cognitive domains are most affected by its use, the measurement instruments used in the studies, as well as the data on the possible improvement in the mood of individuals who participated in the intervention through dance.

## METHODS

The present systematic review used the Preferred Reporting Items for Systematic Reviews and Meta-Analyses (PRISMA) to guide its execution and is registered on The International Prospective Register of Systematic Reviews (PROSPERO) under the protocol number CRD42021273912. The present study is exempt from the evaluation of the Research Ethics Committee as it is a systematic review. The study contemplated the electronic literature in the area, including original and peer-reviewed studies. The following databases were consulted: PubMed, Cochrane Library, and SciELO, during the months of March and April 2021. The last search was performed on April 25, 2021.

The search terms were chosen by consulting the MeSH (Medical Subject Headings) for the terms in English and the DeCS (Descritores em Ciências da Saúde) for the terms in Portuguese and Spanish. The selected terms, and their variations, were aged, cognition, cognition disorders, dance therapy, and senior dance combined as follows:

1. Aged, cognition, dance therapy;
2. Aged, cognition, senior dance;
3. Aged, cognition disorder, dance therapy; and
4. Aged, cognition disorder, senior dance.

The same search strategy was used across all scientific bases with the three languages: “Aged” AND “Cognition” OR “Cognition Disorders” AND “Dance Therapy” OR “Senior Dance.

Included studies must be longitudinal, written in English, Portuguese, and/or Spanish, and published within the past 10 years. Study participants must be over 60 years of age and have cognitive impairment of any degree of severity. Studies must have dance as an intervention method, regardless of the duration of the intervention. Furthermore, another important inclusion criterion was the presence of cognitive assessment, in order to measure the effectiveness of the intervention on the participants’ cognitive performance. As a secondary objective, it was also verified whether the mood of the participants was assessed, although mood was not, by itself, an inclusion criteria. The exclusion criteria adopted were whether the study was a systematic reviews, meta-analysis, theses, and monographs. Thus, these were excluded from the present systematic review.

The research and evaluation process was carried out by two independent reviewers, following the method mentioned above. Any possible discrepancies were discussed and reviewed among the two reviewers. If there was still disagreement, a third reviewer was asked to decide whether or not to include the study. All publications found were included in a spreadsheet, and the extracted data were compared between the two reviewers before the writing of this review.

## RESULTS

**Figure 1:**
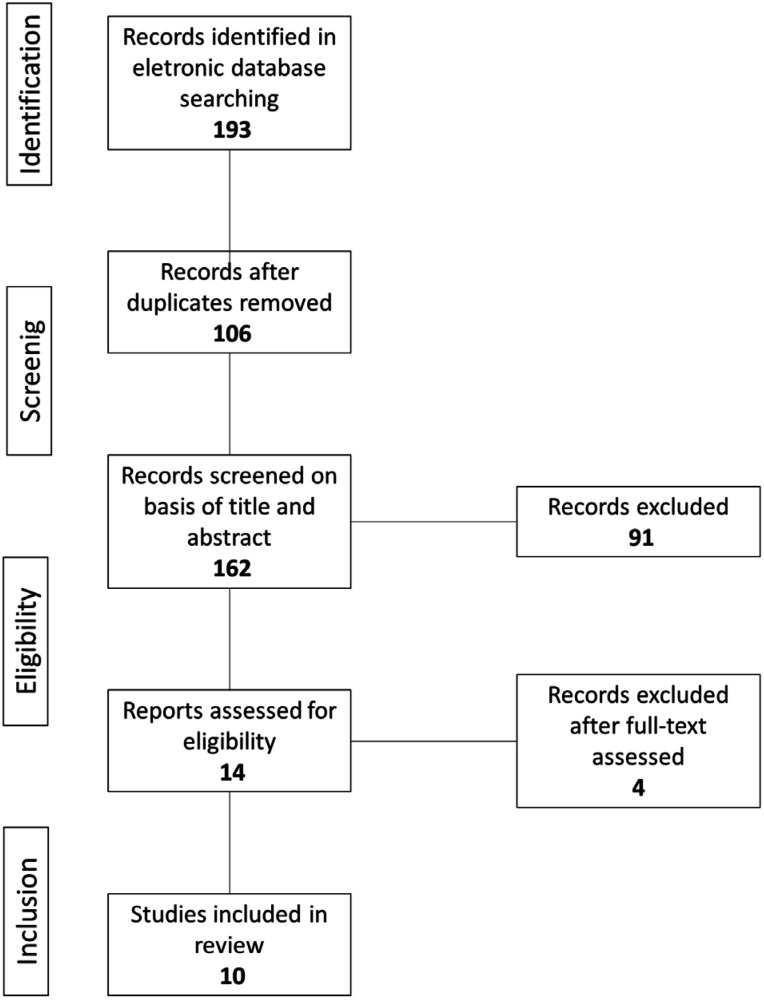
Flowchart of review process and study selection.

In total, 193 papers were found. Of these, 87 were excluded due to duplicity. Of the remaining 106 papers, an analysis of titles and abstracts was performed, taking in consideration the inclusion and exclusion criteria. As a result, 43 papers were excluded due to the age of the participants, 18 because they were not longitudinal studies, 8 for not using dance as an intervention, 12 studies for not carry out cognitive assessment, and 10 studies did not have a sample with cognitive impairment. In total, 91 studies were excluded at this stage, and 14 papers remained eligible to be read in full. This process is shown in the flowchart in Figure 1.

Of the 14 approved publications, 1 was excluded due to impossibility of access by the authors^17^, 1 was excluded because it did not present results^18^, 1 was excluded because it was a pilot study^19^, and 1 was excluded because it did not explain whether the sample had cognitive impairment^20^.

Thus, of the 14 publications originally covered, only 10 met the inclusion criteria and described the effects of dance therapy on the cognition of older people with cognitive impairment. Of these 10 scientific publications, 9 were randomized controlled trials (RCT) and 1 was a quasi-experimental study.

Regarding the location of interventions, four studies were carried out in Europe^21–24^, one in South America^25^, one in North America^26^, and four in Asia^27–30^. In total, the 10 studies had 890 participants, who were divided into groups with dance intervention (IG) and 12 control groups (CG). Other types of intervention used for the CG included varied physical activity (two groups)^22,30^, activities with a psychoeducational group (two groups)^26,29^, playing musical instruments (one group)^29^, and relaxation exercise (one group)^27^. In addition, six CG chose to maintain only their regular daily activities throughout the study period, therefore not performing extra activities^21,23–25,28,30^. Of the 890 participants, 412 received specific dance intervention.

Regarding the cognitive performance of the participants, five studies had samples composed of participants with MCI^21,22,27,29^, two included participants with the diagnosis of dementia^25,30^, and one had participants with Parkinson’s disease^26^. Moreover, some studies had a mixed sample of healthy aged participants and others with cognitive impairment^23,24^. These papers were included in this review, since the inclusion of participants with MCI in the sample justifies their participation.

The interventions had different durations and weekly frequencies; five studies had sessions twice a week^21,22,26,27,30^, while four studies had sessions three times a week^23–25,28^. Of the 10 studies analyzed, only 3 did not have 60 min sessions, opting for 35, 50, and 90 min, respectively^25,26,28^, with the duration of the intervention varying between 6 and 40 weeks^21,27^, with 12-week interventions being more performed, found in 4 studies^22,25,28,30^.

After analyzing the chosen intervention methods, it is noteworthy that most studies — six of them — opted for a warm-up period at the beginning of the sessions and a moment of along with a cool-down (relaxing) at the end, ranging from 5 to 20 min^21,22,25–28^. The rhythms and styles of dance used throughout the sessions were also diversified. Three studies used ballroom dancing^21,25,29^, one study presented the poco-poco dance, a traditional dance from Indonesia^27^; two studies worked with aerobic dance^22,28^; one study approached the tango^26^; two studies performed various rhythms over the weeks^23,24^, and, finally, one study described the dance style only as an adaptation of a well-established dance program in the Chinese community^30^. The programs were conducted by different professionals, including physiotherapists^22,27^, dance-movement therapist^30^, dance instructors^21,26,28,29^, and other professionals^23–24^.

When it comes to the quality and validity of the studies included in this review, a few factors may also be worth mentioning.

First, most studies applied a method of randomization of participants. In fact, 8 of the 10 studies explicitly stated that participants were randomly allocated between IG or CG. The two studies that did not meet this criterion were excluded because:

1. They did not specify whether the participants were randomized^26^ or
2. They stated that there was no randomization and/or blinding involving the allocation of participants^27^.

Some studies did not explicitly mention whether allocation secrecy was a factor taken into account during the participant randomization process; in contrast, 5 of the 10 studies used this method, stating that a third independent evaluator participated in the randomization of the participants^21,22,24,28,29^. The study that did not randomize the participants did not present an evaluation regarding allocation confidentiality^27^, and the rest of the studies did not mention whether randomization was performed blindly or openly.

The studies that blinded researchers to the sample groups could only be categorized as single-blind studies. This is due to the fact that, due to the very nature of the intervention, there is no way for the dance program instructor not to know the allocation of participants, since the dance group is, necessarily, the IG. However, one strategy adopted to alleviate these effects was the use of evaluators who did not have prior knowledge of participant allocation to assess pre- and post-intervention rates in people who completed the program. Some studies implemented this strategy^21,22,28–30^, while others did not mention any type of information about this criterion^23–25,27^, and one study reported keeping only some raters blinded instead of all of them^26^. For reasons similar to those of the instructors, in most cases, the participants also had no way of being blinded in relation to their allocation to the groups. In an attempt to mitigate this situation, one study did not inform participants about the study hypothesis or outcome measures^21^.

Few studies described, in detail, the way researchers dealt with participant losses or exclusions. However, those who did present at least one of the following information: mean adherence to the program, percentage of losses, or attrition bias rates^21–25,28–30^. Some studies excluded losses from the final analysis. However, one study stated that although some participants dropped out of the program during the course of the study, they were still included in the analysis^29^. It is unclear whether these losses were included in the CG analysis or whether they were incorporated into the groups in which originally belonged. Another study stated that no participant withdrew from the program during the intervention^25^.

Before starting the intervention, some studies also calculated the required number of participants in each group so that statistical significance could be achieved; therefore, the researchers took this information into account when planning the intervention and when estimating the total number of participants needed, with the objective of preserving the methodological integrity of the study in possible and eventual cases of loss of participants^22,24,28^.

Concerning the instruments used to assess cognition, the studies showed a considerable diversity in tests and tools. To assess global cognition, the instruments most used were the *Mini-Mental State Examination* (MMSE) and the *Montreal Cognitive Assessment Test* (MoCA). Memory was assessed by an incredibly high number of different tests, the most common being the *Logical Memory* subtest from the *Wechsler Memory Scale-III* (WMS-III). Other instruments included *Rivermead Behavioral Memory Test* (RBMT), *Rey Auditory Verbal Learning Test* (RAVLT), and*Taylor Figure Test*. To evaluate executive function and visuospatial abilities, the *Trail-Making Test* (parts A and B) was the most used instrument, followed by *Judgment of Line Orientation*. *Tower of Hanoi* (ToH), *Five-Point Tes*t, *Rey Osterrieth Complex Figure Test,* and *Taylor Figure Test* were also mentioned. Regarding attention, the most used tests were the subtests *Digit Span* and *Symbol Search* from the *Wechsler Adult Intelligence Scale-III* (WAIS-III), although more tests were also used. In language assessment, the tests chosen were *Verbal Fluency F-A-S* (FAS) and *Boston Naming Test*.

Overall, the studies presented positive results regarding the improvement in cognitive performance as a result of dance intervention. A brief summary of the main findings can be found in Table 1. Memory seems to have been the cognitive function that obtained the most benefits from this kind of intervention; 5 of the 10 studies showed an increase in the participants’ performance in the instruments that assess memory^21,22,24,29,30^. More specifically, verbal memory seems to have been the one more commonly affected^21,22,29,30^. Even though Kropacova et al.^24^ findings did not find statistical significance among IG and CG, the subtle improvement may be indicative of a positive effect of dance in this cognitive function.

**Table 1:** Main results of included publications.

Authors (year/country)	Title	Number of participants/control group	Dance styles and musical rhythms	Tools	Main results
Adam et al.^27^ (2016/Malaysia)	Effectiveness of a combined dance and relaxation intervention on reducing anxiety and depression and improving quality of life among the cognitively impaired elderly	n=84 The CG had relaxation sessions, covering 40 participants of the 84 individuals in the study	A combination of *poco-poco* dance and relaxation exercises	Mini-Mental State Examination (MMSE) Hospital Anxiety and Depression Scale (HADS)	As a result of the 6-week dance intervention, there was a significant difference in the levels of global cognition, anxiety, and depression, measured by the MMSE and HADS, respectively
Bisbe et al.^22^ (2020/Spain)	Comparative cognitive effects of choreographed exercise and multimodal physical therapy in older adults with amnestic mild cognitive impairment: randomized clinical trial	n=31 The comparative group was dedicated to physical exercise, and 14 participants out of the 18 who were initially drafted completed the PE program	Choreographed aerobic dances that used a variety of music styles: salsa, rock, rumba, pop, and jive	MMSE Word list learning from the Wechsler Memory Scale – Third Edition (WMS-III) Visual memory subtest of the Repeatable Battery for the Assessment of Neuropsychological Status (RBANS) Trail-Making Tests A and B (TMT-A and TMT-B) Letter Verbal Fluency (LVF) and Category Verbal Fluency (CVF) Boston Naming Test (BNT) Judgment of Line Orientation	The dance group showed greater benefits with regard to verbal recognition memory, at statistically significant rates. Furthermore, it also showed improvements in delayed visual memory
Borges et al.^25^ (2018/Brazil)	Effects of dance on the postural balance, cognition and functional autonomy of older adults	n=60 30 people were included in the CG, which adopted a life-as-usual approach	The program was based on ballroom dance and included styles such as foxtrot, waltz, rumba, swing, samba, and bolero	HADS MMSE	From the results found after 12 weeks of intervention with dance, it could be observed that the individuals in the experimental group showed an improvement in the mental state exam when compared to the CG, which did not undergo any intervention
Doi et al.^29^ (2017/Japan)	Effects of cognitive leisure activity on cognition in mild cognitive impairment: results of a randomized controlled trial	n=172 117 out of the 172 participants were allocated to groups that were not the dance program; 67 went to a passive CG, and 54 were allocated to a music group	Ballroom dance, including salsa, rumba, waltz, cha-cha, blues, jitterbug, and tango	Story memory and word list memory tests from the National Center for Geriatrics and Gerontology Functional Assessment Tool MMSE Tablet version of the TMT-A and TMT-B	Dance program participants demonstrated an improvement in memory function (more pronounced in patients with amnesic MCI) and in global cognition. No significant differences were found in attention or executive function
Ho et al.^30^ (2020/China)	Psychophysiological effects of dance movement therapy and physical exercise on older adults with mild dementia: a randomized controlled trial	n=166 Participants in the comparative groups were randomized to either exercise or a waitlist control; 56 participants were allocated to the exercise group and 55 were included on the waitlist	According to the authors, the intervention was a modified program from an established dance/movement therapy program, applied within the Chinese population over the past decade	Full object memory evaluation Digit Span Test from the Wechsler Adult Intelligence Scale (WAIS) TMT-A and TMT-B De Jong Gierveld Loneliness Scale Geriatric Depression Scale (GDS) Visual Analogue Mood Scale	Improvements were observed in the domains of verbal memory and verbal fluency. Furthermore, the rates of depression, loneliness, and negative mood decreased. With the exception of memory and loneliness, the benefits observed were mostly qualitative and did not reach statistical significance
Kropacova et al.^24^ (2019/Czech Republic)	Cognitive effects of dance-movement intervention in a mixed group of seniors are not dependent on hippocampal atrophy	n=99 The CG, which was a life-as-usual CG, included 50 participants	Mentioned dances were Irish country, African dance, Greek dance, and tango	Montreal Cognitive Assessment Test (MoCA) Taylor Figure Test Logical memory from WMS-III Symbol search from Wechsler Adult Intelligence Scale – Third Edition (WAIS-III) Digit span from the WAIS-III Tower of Hanoi Five-Point Test Judgment of Line Orientation Test Taylor Figure Test MMSE MoCA Rivermead Behavioral Memory Test (RBMT) Verbal Fluency F-A-S Test (FAS) TMT-B Rey Osterrieth Complex Figure Rey Auditory Verbal Learning Test Test of Everyday Attention (TEA) Neuropsychiatric Inventory (NPI) GDS Beck Depression Inventory (BDI) Hamilton Scale for Depression Perceived Stress Scale (PSS) Beck Anxiety Inventory (BAI)	There was no significant difference among the intervention and the CG. However, slight improvements were observed in the intervention group when compared to its performance at baseline. More specifically, the areas identified as being the most benefited were memory and executive function
Lazarou et al.^21^ (2017/Greece)	International ballroom dancing against neurodegeneration: a randomized controlled trial in Greek community-dwelling elders with mild cognitive impairment	n=129 63 participants out of 129 were allocated to the CG, which received no intervention	Ballroom dance, expressed through styles such as: tango, waltz, Viennese Waltz, foxtrot, tumba, cha-cha, swing, salsa, merengue, Disco-Hustle, and Greek traditional ballroom dancing	MoCA Reverse Corsi Blocks Brooks Spatial Task	The intervention group demonstrated a significant improvement in the domains of global cognition, memory, alternating attention, verbal fluency, executive function, visuospatial skills, processing speed, and learning. The mood of the participants also improved. Comparatively, the CG did not show these benefits
McKee and Hackney^26^ (2013/the United States)	The effects of adapted tango on spatial cognition and disease severity in Parkinson’s disease	n=63 Out of the 9 participants allocated to the CG, which underwent education classes, 8 finished the program	Tango	MoCA Taylor Figure Test Logical memory from WMS-III Digit span from the WAIS-III Symbol search from the WAIS-III Five-Point Test Tower of Hanoi Taylor Figure Test Mississippi Aphasia Judgment of Line Orientation Screening Test BDI-II MoCA Logical memory from WMS Symbol Digit Modalities Test (SDMT) TMT-A and TMT-B Forward and Backward Digit Span Task GDS-15	Dance group participants showed qualitative improvement in spatial cognition and disease severity when compared to their performance at baseline, although it did not reach statistical significance when compared to the CG
Rektorova et al.^23^ (2020/Czech Republic)	Brain structure changes in nondemented seniors after six-month dance-exercise intervention	n=62 31 participants assigned to the CG, which was passive, completed the trial	The authors mentioned that the topic of the program was “Travelling Around the World”, which was constituted by dance styles like folk, country, African, Greek, and tango dancing”	MoCA Taylor Figure Test Logical memory from WMS-III Digit span from the WAIS-III Symbol search from the WAIS-III Five-Point Test Tower of Hanoi Taylor Figure Test Mississippi Aphasia Judgment of Line Orientation Screening Test BDI-II	The intervention produced a slight improvement on executive function. The sample was mixed, including healthy older people and those with MCI. The results were more pronounced in older persons with MCI. In terms of neuroimaging, an increase in cortical thickness induced by the intervention was obtained compared to the CG in the areas of the inferior right temporal, fusiform, and lateral occipitotemporal gyrus
Zhu et al.^28^ (2018/China)	Effects of a specially designed aerobic dance routine on mild cognitive impairment	n=54 The CG consisted of 31 participants and underwent a life-as-usual approach	Aerobic dance	MoCA Logical memory from WMS SDMT TMT-A and TMT-B Forward and Backward Digit Span Task GDS-15	Participants in the intervention group demonstrated significant improvements in memory, processing speed, and global cognition when compared to their baseline performance. These improvements attenuated after 6 months. The CG showed improvements in global cognition, but these benefits were not maintained over time

CG: control group; MMSE: Mini-Mental State Examination; HADS: Hospital Anxiety and Depression Scale; WMS: Wechsler Memory Scale; RBANS: Repeatable Battery for the Assessment of Neuropsychological Status; TMT: Trail-Making Tests; LVF: Letter Verbal Fluency; CVF: Category Verbal Fluency; BNT: Boston Naming Test; MCI: mild cognitive impairment; WAIS: Wechsler Adult Intelligence Scale; GDS: Geriatric Depression Scale; MoCA: Montreal Cognitive Assessment Test; RBMT: Rivermead Behavioral Memory Test; FAS: F-A-S Test; TEA: Test of Everyday Attention; NPI: Neuropsychiatric Inventory; BDI: Beck Depression Inventory; PSS: Perceived Stress Scale; BAI: Beck Anxiety Inventory.

Global cognition, measured by the MMSE and/or MoCA, was also frequently improved as a result of the intervention^21,25,27–29^. Even though it was less frequent, some of the studies also found improvements in the executive function of the participants^21^; in some cases, these benefits were more qualitative than statistically significant^23,24^. Other functions such as language, visuospatial abilities, processing speed, and learning were sporadically quoted by only a few studies and not consistently.

Among the 10 studies analyzed, 6 used instruments to assess mood, 2 used the *Beck Depression Inventory II* (BDI)^21,23^, 2 other used the *Hospital Anxiety and Depression Scale* (HADS)^22,27^, and 3 studies opted for the *Geriatric Depression Scale* (GDS)^21,28,30^. In addition, four studies had as exclusion criteria people with major depressive disorder.

It is worth mentioning that three studies presented significant results in terms of improvement in depressive symptoms, one of which used HADS^27^. Doi et al.^29^ utilized GDS with instruments that assessed loneliness and negative mood (i.e., *Jong Gierveld Loneliness Scale and Visual Analogue Mood Scale*), obtaining positive results in all of these aspects. Lazarou et al.^21^ not only used the GDS but also opted for BDI, achieving improvement in both tests.

On the analysis of the limitations found in the studies, it is possible to conclude that the three most common limitations were participant dropout during the study^22,25^, the small sample size^22,27^, and the lack of neutral CG^22,27^. In relation to the absence of a neutral CG, one of the studies pointed out as a limitation the inexistence of a CG that did not perform any type of activity. Furthermore, a study points to the participation bias that may occur due to the sample’s predisposition to perform physical activities^30^. To avoid this bias, one possibility is to compare individuals with similar levels of physical activity^22^. Regarding participation bias, the number of dropouts was noticeable as pointed out in the limitations, and aiming to limit selection biases, one study chose to use the random allocation and blinding algorithm.

## DISCUSSION

The present systematic review is the only one up to the point of writing this paper to present an analysis of the evidence obtained in dance interventions with older people with cognitive impairment, including diagnoses such as Alzheimer’s disease, MCI, and Parkinson’s disease^25–27^: Other reviews analyzed the impact of dance in healthy individuals, or otherwise with only one kind of impairment^16–21,31^. And so, this study aimed to discuss what are the impacts of dance therapy on older people with different kinds of cognitive impairment.

The interventions performed had a wide variation in the duration of the stimulation time, but the studies that proposed sessions over 6 weeks and those that performed stimulation with a maximum of 40 weeks found significant results of cognitive improvement. The widely varied styles of dance present in the studies can be considered a representation of the cultural expression of dance; along with music, it is widely accepted that dance has a universal nature and takes different formats depending on the environment and society. But even with all this diversity, they all produce similar outcomes by stimulating choreographed and rhythmic body movements accompanied by musical harmony, producing the integration of brain regions that are activated during spatially patterned bipedal and rhythmic movements^32^.

Regarding cognition, most studies showed positive results. The effect of physical exercise on the cognition of older people seems to be well documented in the literature, with significant but modest results in those individuals with MCI. The evidence is a little more controversial when it comes to people with dementia^33^. When it comes specifically to the influence of dancing on the brain, previous reviews have pointed out that this type of intervention plays an important role in increasing hippocampal volume as well as gray matter in the prefrontal and parahippocampal gyrus; the integrity of the white matter also seems to benefit^34^. Concerning cognitive aspects, the literature points to improvements due to dance interventions in the cognitive state of participants with Alzheimer’s disease^16^. However, as mentioned above, when it comes specifically to older people with dementia and cognitive impairment, simultaneously, so far there is no systematic review in which the evidence is conclusively covered.

Of all the studies, all IG, without exception, showed improvements in — or, at least, the preservation of — their cognitive abilities. Global cognition, measured by the MMSE and MoCA, seems to be the domain that most benefited from the dance intervention^21,25,27–29^, along with verbal memory^21,22,24,29,30^. This is in agreement with the findings of Wu et al.^35^, in which it is stated that the global cognition of individuals with MCI seems to be more susceptible to dance intervention than that of healthy older people. Santos et al.^36^ also denoted benefits associated with the memory of older people who participated in a Senior Dance program.

Improvements in executive function were also found through the analysis in our review; however, these changes were mostly discrete and not statistically significant in general^23,24^, with the exception of one study^21^ that achieved statistical significance of p<0.05. According to a review carried out by Wang et al.^37^ about the influence of body-mind exercise on the cognition of people with cognitive impairment, the effect of the intervention on the executive function of the participants was also not significant. In this review, other domains such as language, attention, visuospatial abilities, learning, and processing speed were identified as benefiting from the intervention. However, these benefits were inconsistently achieved — many of the improvements were found only in one study — which is a result that cannot be considered robust enough to allow us to draw a causal relationship among dance intervention and improvement in cognition. Although the results were promising, future studies are needed to better assess these issues.

Therefore, dance intervention has shown to be a promising strategy for cognitive improvement in people with MCI and dementia with regard to memory (specially verbal memory) and global cognition.

The literature already recognizes some positive effect of dance on mood, specifically with regard to anxiety and depression, which suggests a potential in dance interventions as a possible and important therapeutic complement in the treatment of these pathologies^36^. The results found in this review indicate that, of six studies in which mood was assessed pre- and post-intervention, only three did not achieve positive results. Two of these three studies used major depressive disorder as an exclusion criteria^22,23^, and the last one^28^ found that the mood of participants allocated to the CG also improved, but depression scores did not reach a significant difference among the CG and IG. The three studies that found benefits did not exclude participants who had depressive symptoms. This suggests that people with depressive symptoms may show benefits from the dance intervention in mood performance as well. It is important to emphasize that the current evidence is not strong enough to establish a causal relationship and that future studies on the subject are still very much needed. In the meantime, based on the results obtained, it is possible to conclude that dance interventions can be useful as a therapeutic tool to improve the mood of people with cognitive impairment and depressive symptoms.

Interestingly, the type of intervention chosen — that is, dance style and/or musical rhythms — did not significantly affect depressive symptoms after the end of the intervention programs. All studies that obtained improvement in depressive symptoms did not associate it with the type of music chosen, which seems to suggest that the dance style has a low influence on the results related to mood performance. Especially in comparison with other variables such as motivation, participation, and adequate instructions, among others, which in turn were associated with improvement in depressive symptoms. It is necessary to reinforce that these data are still limited to affirm or deny a causal relationship between these variables.

Studies have limitations that are regularly found when it comes to research involving older participants, due to the peculiarities of working with this population. The limitation most frequently cited by the authors was the struggles in adherence to the program, which, in result, had a direct impact on the sample size^22,25,27^. It is also important to note that future research is needed to assess possible differences in the effectiveness of the intervention arising from the duration of the dance program, assess whether these improvements withstand the passage of time, and determine whether there is any correlation among the duration of the intervention and the strength of the results when it comes to long-term testing. It is also important to mention the need for further investigation into whether dance interventions are able to prevent or delay cognitive impairment and distinguish the impact of dance according to different kinds of impairment.

This present review also had its fair share of limitations; in the first place, the authors could not have access to the study conducted by Dominguez et al.^17^, even though it met all the chosen inclusion criteria. Furthermore, even though the vast majority of studies written in English were performed in countries that had other native languages, the lack of access to papers in other languages also stands out as a limitation, since they could have contributed to expanding the comprehension of impacts of dance in relation to sociocultural influences. In addition, the methodology employed in this review to investigate the studies did not use statistical analysis of the results, nor did it use qualitative analysis protocols, which can also be perceived as a limitation.

Thus, it is still necessary that future studies elucidate some gaps present in the current knowledge on the subject, such as the level of impact attributed to the dance intervention regarding:

1. The cognitive profile of the sample,
2. The chosen rhythm,
3. The professional training of the person responsible for carrying out the intervention, and
4. The degree of cognitive and motor performance required by the choreography.

Furthermore, there are gaps in the literature regarding theoretical-conceptual models of sensory, cognitive, and motor stimulation that take into account differences in cognitive status and functional performance. Future studies should also be dedicated to the creation of dance intervention protocols for the older people, aimed at specific cognitive and motor profiles; finally, the methods of application and the activities performed should also be better explained in future publications.

The recent growth of the older population is accompanied by the expansion of the occurrence of pathological states characterized by cognitive impairment, such as MCI and dementia. Consequently, the search for effective treatments to manage and delay the progress of those syndromes is still very present. Considering the data obtained in the present review, it is possible to claim that the use of dance therapy is a viable alternative as a nonpharmacological treatment for people with cognitive impairment of any kind. Since dance programs are a noninvasive and low-cost strategy, they have great applicability to the population. Through consideration of electronic literature, evidence suggests that programs of dance intervention offer relevant benefits in certain cognitive domains, such as global cognition, memory, and executive function. Other cognitive functions, such as attention and language, still need to be better investigated in future experiments in order to verify the effectiveness of this kind of intervention.

Regarding the mood of the participants, three of the six studies showed effective benefits in the depressive symptomatology of aged people with cognitive impairment, which suggests the potential effectiveness of dance as a therapeutic option in this aspect as well. Therefore, considering the data obtained, it is possible to claim that dance intervention can be a strong ally in the treatment of symptoms arising from cognitive impairment in older people.

As previously mentioned, dance therapy is a relatively low-cost intervention; the only significant expenses would be the monetary resources needed to hire the assistance and expertise of a dance professional who would be able to choreograph the program and coordinate the sessions or, at least, prepare and educate the professionals who apply these sessions. Since this type of intervention clearly does not lose any of its benefits when applied in groups, its practical nature and wide applicability are some of its strongest aspects.

Given the above, the implementation of this type of intervention in primary health care is possible and probably quite feasible. The benefits of physical exercise are well known to most, if not all, and the older population is constantly encouraged to maintain a healthy lifestyle and exercise; it is not a stretch to suggest that health professionals could also encourage these people to participate in dance programs when possible, especially if they are available in their own community. Likewise, the creation and organization of those programs should also be achievable in the context of hospitals, health centers, and nursing homes.

Currently, there is no standardized protocol for a cognitive intervention focused on dance therapy. In this sense, it is clear that there is a gap in knowledge and accessibility of the general public regarding a scientifically reliable dance program for older people with cognitive impairment. Future research in this field can, and should, focus on the creation and organization of dance protocols based on the methodology of studies that have managed to gather significant and relevant results, in order to mitigate this situation.
